# Tetrahydrobiopterin Supplementation: Elevation of Tissue Biopterin Levels Accompanied by a Relative Increase in Dihydrobiopterin in the Blood and the Role of Probenecid-Sensitive Uptake in Scavenging Dihydrobiopterin in the Liver and Kidney of Rats

**DOI:** 10.1371/journal.pone.0164305

**Published:** 2016-10-06

**Authors:** Akiko Ohashi, Yusuke Saeki, Tomonori Harada, Masako Naito, Tomihisa Takahashi, Shin Aizawa, Hiroyuki Hasegawa

**Affiliations:** 1 Department of Anatomy, Nihon University School of Dentistry, Chiyoda, Tokyo, Japan; 2 Department of Biosciences, Teikyo University of Science and TechnologyUenohara, Yamanashi, Japan; 3 Division of Anatomical Science, Department of Functional Morphology, Nihon University School of Medicine, Itabashi, Tokyo, Japan; Nathan S Kline Institute, UNITED STATES

## Abstract

Tetrahydrobiopterin (BH_4_) is an essential cofactor of nitric oxide synthase (NOS) and aromatic amino acid hydroxylases. BH_4_ and 7,8-dihydrobiopterin (BH_2_) are metabolically interchangeable at the expense of NADPH. Exogenously administered BH_4_ can be metabolized by the body, similar to vitamins. At present, synthetic BH_4_ is used as an orphan drug for patients with inherited diseases requiring BH_4_ supplementation. BH_4_ supplementation has also drawn attention as a means of treating certain cardiovascular symptoms, however, its application in human patients remains limited. Here, we tracked biopterin (BP) distribution in blood, bile, urine, liver, kidney and brain after BH_4_ administration (5 mg/kg rat, i.v.) with or without prior treatment with probenecid, a potent inhibitor of uptake transporters particularly including organic anion transporter families such as OTA1 and OAT3. The rapid excretion of BP in urine was driven by elevated blood concentrations and its elimination reached about 90% within 120 min. In the very early period, BP was taken up by the liver and kidney and gradually released back to the blood. BH_4_ administration caused a considerable decrease in the BH_4_% in blood BP as an inevitable compensatory process. Probenecid treatment slowed down the decrease in blood BP and simultaneously inhibited its initial rapid excretion in the kidney. At the same time, the BH_4_% was further lowered, suggesting that the probenecid-sensitive BP uptake played a crucial role in BH_2_ scavenging *in vivo*. This suggested that the overproduced BH_2_ was taken up by organs by means of the probenecid-sensitive process, and was then scavenged by counter-conversion to BH_4_ via the BH_4_ salvage pathway. Taken together, BH_4_ administration was effective at raising BP levels in organs over the course of hours but with extremely low efficiency. Since a high BH_2_ relative to BH_4_ causes NOS dysfunction, the lowering of the BH_4_% must be avoided in practice, otherwise the desired effect of the supplementation in ameliorating NOS dysfunction would be spoiled.

## Introduction

Tetrahydrobiopterin (BH_4_) is an essential cofactor for a group of aromatic amino acid hydroxylases [1–4]. Inherited BH_4_ deficiencies are characterized by hyperphenylalaninemia and defective biosynthesis of classic monoamines such as dopamine, noradrenaline, adrenaline, as well as serotonin. BH_4_ therapy using (6R)-L*-erythro*-5,6,7,8-tetrahydrobiopterin dihydrochloride (6RBH_4_) has been very successful in replacing peripheral BH_4_, but not BH_4_ in the brain. A guide for the therapeutic use of BH_4_ is available [5]. This compound ameliorates hyperphenylalaninemia in cases of inherited BH_4_ deficiency [6, 7] and benefits patients with BH_4_-responsive phenylketonuria [8]. The most common use of 6RBH_4_ to date is as an orphan drug for these inherited diseases [9–11]. Once 6RBH_4_ is administered, it replaces innate BH_4_ and is integrated through endogenous metabolic pathways, similar to the intake of vitamins. However, like most drugs or supplements, large amounts of exogenous BH_4_ bypass endogenous pathways and are eliminated in urine [12, 13] and feces [14], therefore it is desirable to minimize this useless exclusion.

We previously observed that 6RBH_4_ administration raised tissue BH_4_ levels, however, with a concomitant rise in 7,8-dihydrobiopterin (BH_2_) [12, 13]. Urinary excretion was suggested to be the process most responsible for the rapid loss of exogenous BH_4_. Furthermore, we demonstrated that urinary excretion of administered 6RBH_4_ at a pharmacological dose predominantly involved secretion across renal tubular epithelium, distinct from glomerular filtration. We observed that the tubular secretion was almost completely suppressed by cyclosporin A (CsA), an inhibitor of transporters with broad specificity in excretion of xenobiotics and metabolic wastes.

In order to obtain greater insight into the renal exclusion of BH_4_ and the relative increase in dihydrobiopterin (BH_2_), in this study, we analyzed the systemic distribution of biopterin (BP), the sum total of BH_4_ and BH_2_ (BP = BH_2_ + BH_4_), in the presence of probenecid (Pbc), a potent inhibitor of uptake transporters with a broad specificity [15]. We confirmed the active role played by the renal secretion of BP in the heavy loss of exogenous BH_4_. Furthermore, we revised the role of the salvage pathway of BH_4_ biosynthesis in scavenging BH_2_ in terms of its passage through the liver and kidney under the conditions of 6RBH_4_ administration.

## Materials and Methods

### Chemicals

6RBH_4_ was donated by Suntory (Asubio Pharma, Kobe, Japan). 6RBH_4_ (BH_4_•2HCl) was dissolved at 10 mg/mL in 0.9% NaCl before the start of the BH_4_ administration experiment. The BH_4_ solution was neutralized by 0.1 M Na_2_HPO_4_ and saline to prepare a 5 mg/mL concentration immediately before injection. Probenecid (Pbc: *p*-(dipropylsulfamoyl)benzoic acid) and cyclosporin A were obtained from Sigma-Aldrich (St. Louis, MO). Pbc was dissolved by dropwise addition of 2M NaOH, followed by the addition of N-2-hydroxyethylpiperazine-N'-2'-ethanesulfonic acid (HEPES) to neutralize the solution and adjusted the concentration to 40 mg/mL. CsA was dissolved in ethanol, then diluted in 9 vol of olive oil (final concentration, 5 mg/mL). “Gall Powder®” was purchased from Wako Pure Chemical Industries (Osaka, Japan).

### Ethics statement

The animal experiments were conducted in accordance with the ethical guidelines of the Teikyo University of Science and Technology Animal Experimentation Committee, and guidelines of the Japanese Pharmacological Society. The protocol was approved by the Teikyo University of Science and Technology Animal Experimentation Committee (Permit Number: B-08005). All efforts were made to minimize suffering. Rats (SD: Sprague Dawley) were obtained from Japan SLC (Hamamatsu, Japan) and bred in our SPF-grade laboratory. The animals were maintained on a constant 12-h light-dark cycle at 21–24°C at 40–60% humidity and were provided with ordinary laboratory chow and sterile tap water ad libitum. For deep but a short period of anesthesia, rats were administered sodium pentobarbital (30 mg/kg for light anesthesia, 50 mg/kg for deep, both i.p.). For experiments taking a longer time (4 ~ 6 hours), rats were anesthetized with an i.p.-administered cocktail of pentobarbital (45 mg/kg), atropine (0.05 mg/kg), dimorpholamine (Theraptique, 1.2 mg/kg), and xylazine (9.5 mg/kg) as described previously [12, 13]. The anesthetized rats were kept on a warm gel pad (40°C) and given a 1/3 to 1/2 dose every hour to maintain them under anesthesia.

### Experimental procedure

All rats (8–10 weeks old, male and female random mix) were loaded with 6RBH_4_ (5 mg/mL) at a dose of 5 mg/kg (i.v.) under anesthesia. Administration of Pbc (200 mg/kg, i.p.) or CsA (10 mg/kg, i.p.) was performed 30 min prior to 6RBH_4_ administration. Experiments were carried out by means of two different protocols; (A) by sequentially collecting blood, bile and urine from individual rats under long-lasting anesthesia on a warm gel pad, and (B) by removing the liver, kidney and brain after sacrificing the respective rats under the deep anesthesia at specified times after 6RBH_4_ administration.

(A) Rats were intraperitoneally administered Pbc (“BH_4_ + Pbc”, n = 5) or saline as a control (“BH_4_ alone”, n = 7) and were then anesthetized. All rats were given 6RBH_4_, then subjected to sampling at specified times as previously described [12, 13], except that bile was also taken and the animal was given saline containing 2% bovine bile powder (w/v), at a rate of 0.9 mL/hr, which was administered into the intestinal duct. The bovine bile powder did not contain any measurable BH_2_ or BH_4_. In the following sample acquisition, special care to ensure minimal oxidation of BH_4_ was taken throughout the following procedure. In brief, the abdomen was opened and cannulae were set in place for collecting bile and urine. The blood samples were drawn from the tail vein into a heparin-coated capillary containing a solution of 0.1 M ascorbic acid (less than 5% by volume), and they were then subjected to differential iodine oxidation for BP determination. The urine was collected at intervals from the bladder through an indwelling needle connected to an airtight syringe in which the dead space had been filled with saline containing 50 mM each of ascorbic acid and EDTA (pH 6.8). The urine was immediately mixed with 9 volumes of 10 mM HCl containing 5 mM antioxidant. The aliquots were then subjected to quantitation of BP and creatinine. BP excretion was expressed as BP/creatinine (μmol/mg creatinine) based on the BP concentration (nmol/mL) divided by the time-matched creatinine level (μg/mL). In calculating the cumulative total, the amounts of BP in each fraction were added. Calculations were based on the BP concentration (nmol/mL) multiplied by the respective urine volume (mL) produced between each sampling time point regardless of the creatinine concentration. The bile was also collected from individual rats through a cannula in the bile duct and was immediately subjected to BP determination. The cumulative BP amount was calculated by adding up all samples measured.

(B) Rats were treated with 6RBH_4_ and drugs (Pbc and CsA), or saline as a vehicle control, and allowed to move freely after recovery from the light anesthesia. In the Pbc experiment, animal used numbered n = 4 each for the respective administration of “BH_4_ + Pbc”, and the vehicle control “BH_4_ alone”. In the CsA experiment, the numbers of rats for groups of “BH_4_ + CsA” and “BH_4_ alone” were n = 5, respectively. At 0, 30 and 120 min after 6RBH_4_ administration, blood samples were taken from the abdominal vein into a syringe in which the dead space was replaced with saline containing 50 mM each of ascorbic acid and EDTA (pH 6.8). Each blood sample was divided into three portions; one served as a “whole blood” specimen, the second was for the hematocrit determination, and the third was centrifuged (10,000 rpm, 4°C, 5 min) to separate red blood cells (RBCs) from the plasma. The whole blood, plasma, and RBCs were separately subjected to differential iodine oxidation for the BP determination described below. The results were expressed as the amount of BP per unit volume of the original blood sample (plasma + RBC, mL) based on the hematocrit value. The kidneys, liver, and whole brain were removed without prior perfusion, rinsed with saline, blotted, weighed, frozen in liquid nitrogen, and then stored at -80°C until BP determination. BP carryover with the blood was ignored in the liver and kidney. However, for the brain, the BP carryover was estimated using an average blood inclusion of 1.2% (v/w), which was determined by a comparison of the heme content of the blood and brain homogenates based on light absorbance at 415 nm [16].

### Determinations

Biopterin was determined essentially according to Fukushima and Nixon [17] and modified as described previously [18, 19]. Animal tissues contain at least 4 chemical species of the reduced biopterin family; BH_4_-4a-carbinolamine (or 4a-hydroxy-tetrahydrobiopterin), quinonoid dihydrobiopterin and 7,8-dihydrobiopterin as well as BH_4_. The Fukushima and Nixon method includes tissue homogenization and extraction of pterin compounds followed by measurement of amount of fully oxidized biopterin after differential iodine oxidation under acid and alkaline conditions. With their method, the biopterin quantity remaining after the acid-oxidation was taken as the total biopterin (BP = “BH_4_” + “BH_2_” + “oxidized biopterin”) and that which remained after the alkaline-oxidation, as 7,8-dihydrobiopterin (BH_2_) plus oxidized biopterin. The amount of oxidized biopterin in extracts without the iodine oxidation was disregarded in this study since the observed amount was too small for the purpose of our work. Accordingly, in this study, the biopterin amount measured after the acid iodine oxidation was taken as the sum of “BH_4_” and “BH_2_” (“BP” = “BH_2_” + “BH_4_”), and that after the alkaline oxidation, as the amount of “BH_2_”. The difference was therefore taken as the “BH_4_” amount. The BH_4_ fraction (BH_4_%; the percentage of BH_4_ in BP) was calculated for each individual sample. The amounts of BH_4_ and BH_2_ were calculated based on molecular weights of 241 g/mol and 239 g/mol, respectively. The molecular mass of the reagent 6RBH_4_•2(HCl) was 315g.

The urinary creatinine concentration was measured using a creatininase-HMMPS method according to the manufacturer’s instructions (L-type Creatinine M; Wako Pure Chemical Industries, Osaka, Japan).

### Data presentation

Data were expressed as means ±S.E. The statistical significance of two groups with non-repeated determination was analyzed by Student’s *t*-test. The significance of difference between determinations at different times with individual animal groups was analyzed by the Paired *t*-test. Significance of difference between three groups was analyzed by Tukey’s test. All the statistical analyses were performed with Pharmaco Basic Ver.15.0.1 (Scientist Co. Ltd., Tokyo). Using results of pterin kinetics, exponential least square approximation was performed with Excel software using Microsoft Office for Mac. For the best fit by a first-order equation, *BP*_*t*_ = BP_0_ • e^−k*t*^, data were plotted on a semi-log scale of y-axis *vs*. time (min) on the linear x-axis, forming straight lines as Ln (*BP*_*t*_) = Ln (BP_0_) − k*t*, where BP_0_ represents the extrapolated initial concentration of BP and *BP*_*t*_ at time *t* (in nmol/mL of bile or μmol/mg creatinine of urine). The slope of the plot, k, yields the fractional rate constant (min^−1^), and gives the corresponding decay half-time, T_1/2_ = Ln(2)/k (min).

## Results

### Rat biopterin levels in the blood, urine and bile after 6RBH_4_ administration

We tracked BP contents and BH_4_ fractions (BH_4_%) in blood, urine and bile after rats were administered 6RBH_4_ (5 mg/kg, i.v.) with or without the Pbc treatment (200 mg/kg, i.p.), denoted hereafter as “BH_4_ + Pbc” or “BH_4_ alone”, respectively. Experimental procedure (A) in the Methods section was used.

#### Blood and urine

**Fig 1:**
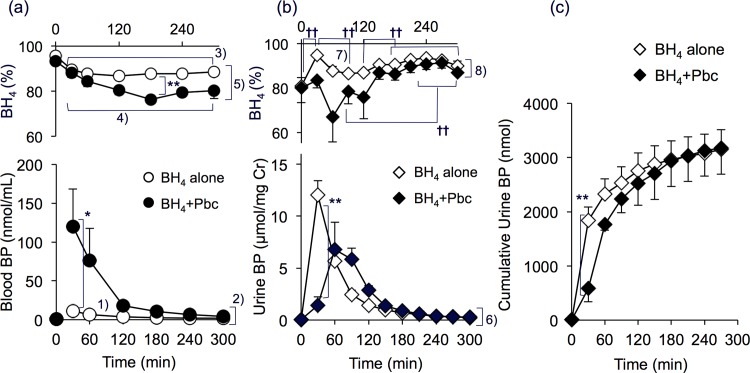
Effect of probenecid on biopterin levels in the blood and urine after 6RBH_4_ administration. Rats were given 6RBH_4_ (5 mg/kg, i.v.) with probenecid (200 mg/kg, i.p., “BH_4_ + Pbc”) or without (“BH_4_ alone”). The blood and urine were collected as in experimental procedure (A) of Materials and Methods. (a) Blood BP (lower panel) and the BH_4_% (upper panel), “BH_4_ alone” (open circles) and “BH_4_ + Pbc” (closed circles). (b) Urinary BP relative to creatinine (μmol/mg creatinine, lower panel) and BH_4_% (upper panel), “BH_4_ alone” (open diamonds) and “BH_4_ + Pbc” (closed diamonds). (c) Cumulative BP content in the urine as a function of time after 6RBH_4_ administration, “BH_4_ alone” (open diamonds) and “BH_4_ + Pbc” (closed diamonds). All data are means ±S.E. of n = 5–7 for “BH_4_ alone” and n = 3–5 for “BH_4_ + Pbc”. Statistical significance: Student’s *t*-test (non-numbered) *P<0.05, **P<0.01; Paired *t*-test (non-numbered) ^††^P<0.01. Numbered significance: (a) 1) “BH_4_ alone”, increased (Paired *t*-test), 0-time *vs*. each data point from 30–300 min, *P*<0.005; in “BH_4_ + Pbc”, all greater than at 0-time, *P*<0.05. 2) “BH_4_ alone” *vs*. “BH_4_ + Pbc” (Student’s *t*-test), *P* = 0.03 at 30 min; *P* = 0.006 at 120 min; *P* = 0.02 at 180 min. 3) “BH_4_ alone”, decreased (Paired *t*-test), 0-time *vs*. each data point from 60–300 min, *P*<0.05. 4) “BH_4_ + Pbc”, decreased (Paired *t*-test), 0-time *vs*. each data point from 30–300 min, *P*<0.003. 5) “BH_4_ alone” *vs*. “BH_4_ + Pbc” (Student’s *t*-test): *P* = 0.05 at 60 min, *P*< 0.004 at each data point from 120–300 min. (b) 6) “BH_4_ alone” *vs*. “BH_4_ + Pbc” (Student’s *t*-test), *P*<0.001 at 30 min; *P*<0.006 at 90 and 120 min; *P* = 0.011 at 150 min. 7) “BH_4_ alone”, increased (Paired *t*-test), 0-time *vs*. 30 min, *P* = 0.005; 0-time *vs*. each data point from 150–270 min, *P*<0.04. 8) “BH_4_ alone” *vs*. “BH_4_ + Pbc” (Student’s *t*-test), *P* = 0.002 at 30 min; *P* = 0.04 at 30 min. (c) “BH_4_ alone” *vs*. “BH_4_ + Pbc” (Student’s *t*-test), *P* = 0.008 at 30 min.

Prior to treatment, the combined BP level in the blood, plasma and red blood cells was 0.57 ± 0.08 nmol/mL and the BH_4_% was 90% to 95%. The BP level increased after receiving 6RBH_4_ “BH_4_ alone”, and gradually decreased to ca. 3.3-fold (*P*<0.005) of the initial level at 300 min (Fig 1A, lower panel). The blood BP content of the “BH_4_ + Pbc” group was much larger than that of the “BH_4_ alone” group. Reflecting this, the AUC_0-120_ of the “BH_4_ + Pbc” group was about 10-fold more than that of the group that received “BH_4_ alone”; 7,580 *vs*. 736 (nmol/mL)•min.

In the “BH_4_ alone” rats, the blood BH_4_% dropped significantly within 30 min and continued to drop over the experimental period (Fig 1A, upper panel). In the “BH_4_ + Pbc” rats, the BH_4_% was substantially decreased but the decrease proceeded gradually; its lowest value of 79% was reached at 180 min and remained low thereafter. The difference in the BH_4_% between the “BH_4_ alone” and “BH_4_ + Pbc” groups was most evident at 180 min (88% *vs*. 79%) (*P* = 0.001).

Urinary excretion of BP (relative to creatinine, μmol/mg Cr) after 6RBH_4_ administration in the presence or absence of Pbc is depicted in Fig 1B. A large amount of BP appeared soon after the 6RBH_4_ mono-loading of “BH_4_ alone” rats, with the peak appearing at around 30 min. On the other hand, the early excretion of BP in the “BH_4_ + Pbc” group at 30 min was strongly suppressed to 11.7% of the amount in the “BH_4_ alone” group (*P*<0.001) while the blood BP level of “BH_4_ + Pbc” rats was 8.5-fold higher than of the “BH_4_ alone” rats (*P* = 0.03) at the same time point. The peak was delayed and appeared between 60 and 90 min. Since glomerular filtration might not be inhibited by Pbc, the observed decrease in BP flow at 30 min was thought to be due to a transporter-mediated process across the tubular epithelial cell layer, consistent with our previous results using CsA [12, 13]. Despite the effective inhibition of the early outflow by Pbc, the outflow appeared to have been delayed, although the gross amount was not reduced. The creatinine-based AUC_0–270_ of the “BH_4_ alone” rats was 731 (μmol/mg Cr)•min, and that of the “BH_4_ + Pbc” rats was 608 (μmol/mg Cr)•min. The gross outflow was substantially completed by 270 min. The cumulative excretion calculated from the urine-volume-based BP reached 3,150 ±360 and 3,170 ±500 nmol in the “BH_4_ alone” and “BH_4_ + Pbc” groups, respectively, representing about 61% of the dose used (Fig 1C). We noted that at as early as 30 min, the BP outflow amounted to 1840 ± 250 nmol in the “BH_4_ alone” group; about 58% of the gross outflow obtained by 270 min. Similarly, it reached 87% of the gross urinary excretion by 120 min. Most of the remainder may have been broken down to pterin or xanthopterin. However, we have no information about the rest of the BP except for an observation with mice about BP movement in to the rumen of the GI tract in our previous study [14]. We observed that a considerable amount of administered 6RBH_4_ appeared in the rumen of mouse small intestine and moved to the caecum, where BP seemed not to be retrieved but essentially metabolized to pterin, a bare pterin-ring compound, by the cecal microflora.

The urinary BH_4_% before receiving 6RBH_4_ was 81 ± 4% (Fig 1B, upper panel). On BH_4_ mono-loading, the percentage had risen to 95 ± 1% at 30 min (Paired *t*-test, *P* = 0.005), presumably representing the amount that reached the kidney bypassing uptake by various organs. Subsequently, the BH_4_% was decreased to 87 ± 2% at 90 min (*P* = 0.01), followed by a gradual increase up to ca. 94% at 240 min (120 min *vs*. each data point from 150–240 min, *P*<0.005). Prior to administration of 6RBH_4_, but 30 min after receiving Pbc, the BH_4_% was 80 ± 7%. It remained relatively low for 120 min and then rose gradually (90 min *vs*. each data point from 210–270 min, *P*<0.005).

#### Bile

**Fig 2:**
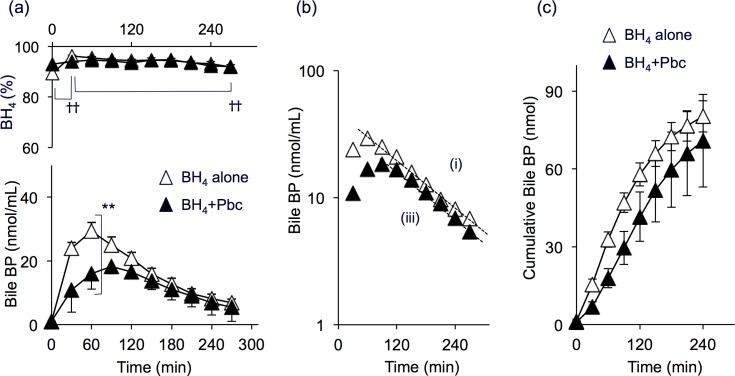
Biliary biopterin excretion after loading rats with 6RBH_4_ with and without prior administration of probenecid. Samples were collected from the same rats as those in Fig 1. The bile was collected from the bile duct, and BP levels and BH_4_ fractions (BH_4_%) were determined following experimental procedure (A) of Materials and Methods. (a) Biliary BP concentration (lower panel) and the BH_4_% (upper panel). One group of rats received “BH_4_ alone” (open triangles), and the other, “BH_4_ + Pbc” (closed triangles). (b) Exponential least-square fitting of biliary BP outflow: “BH_4_ alone”; *BP(t)* = 47.2 • e^−0.007*t*^, correlation coefficient |R| = 0.99. “BH_4_ + Pbc”; *BP(t)* = 37.2 • e^−0.007*t*^, |R| = 0.99. (c) Cumulative BP excretion in the bile. Data in (a) are means ±S.E. of n = 4–6 for “BH_4_ alone” and n = 5–7 for “BH_4_ + Pbc”. Some error bars are hidden behind symbols. Data in (b) are mean values in (a). Paired *t*-test, ^††^*P*<0.01; Student’s *t*-test, **P<0.01.

Excretion of BP in the bile was increased after administration of 6RBH_4_ either with or without Pbc. Levels of the bile BP were consistently higher than BP levels of time-matched blood samples taken from “BH_4_ alone” rats (compare Fig 2A with Fig 1A). At 30 min after 6RBH_4_ administration, for example, the BP level in the bile was 2.2-fold higher than that of time-matched blood (*P* = 0.001). The elevation of the BP content in the “BH_4_ + Pbc” group was 46% less than that of the “BH_4_ alone” group at 60 min (*P* = 0.006). On administration of 6RBH_4_, the biliary BH_4_% was maintained as high as 95% and was not affected by Pbc treatment over the course of the experiment (30–270 min). The bile BP profile appeared to have a limited correlation with that of the blood BP (compare upper panels in Fig 1A and Fig 2A).

Fig 2B presents kinetics based on the same data (average) of Fig 2A. The BP concentration in both groups fitted two nearly parallel lines:

“BH_4_ alone”: *BP(t)* = 47.2 • e^−0.007*t*^ (|R| = 0.99) (i)
“BH_4_ + Pbc”: *BP(t)* = 37.2 • e^−0.007*t*^ (|R| = 0.99) (ii)

The common rate constant of 0.007 min^-1^ denoted a half-decay period of 99 min. This indicated that Pbc caused the BP pool size in bile to become smaller but did not change the rate constant of BP secretion, indicating that Pbc had little effect on the process. The BP pool might represent BP of the liver, the source of this fluid, suggesting that the liver supplied a smaller amount of BP in the presence of Pbc.

As shown in Fig 2C, the cumulative BP amount after administration of “BH_4_ alone” for 240 min was 80.3 nmoles per rat, representing 1.6% of the 6RBH_4_ dose. In addition, the cumulative BP excretion in the presence of Pbc, i.e., that of the “BH_4_ + Pbc” group, was not very different; 70.8 nmoles per rat for 240 min, which was 1.4% of the 6RBH_4_ dose. In either case, the amount of BP excreted into the bile was much less than that into the urine. We previously described excretion of BP in the bile as a route for its entero-hepatic circulation in mice, and a considerable amount of BP secreted in the small intestine was brought to the caecum [14]. In order to prevent bile BP to return to the circulation, we removed all the bile in the procedure (A). If BP was also excreted by the other route such as *via* the intestinal juice under the present experimental conditions, a certain amount of BP was also secreted to rumen of the small intestine and could have moved to the caecum. Possible loss of administered BP by this route remains elusive.

### Organ distribution of biopterin after 6RBH_4_ administration to rats

Organ distribution of BP was examined after 6RBH_4_ administration (5 mg/kg, i.v.) with Pbc (200 mg/kg, i.p.) or CsA (10 mg/kg, i.p.) or without the inhibitor. In this experiment, rats were anesthetized for handling and allowed to move freely after recovery as described in experimental procedure (B).

#### Blood

**Fig 3:**
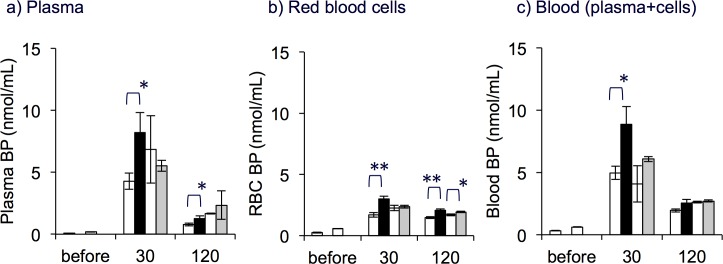
Effect of probenecid and cyclosporin A on the biopterin distribution in blood after 6RBH_4_ administration. Panels show BP levels (nmol/mL) in plasma (a), red blood cells (b), and whole blood (c). The blood was collected as described in experimental procedure (B) of Materials and Methods. The amount of 6RBH_4_ administered was 5 mg/kg, i.v. (“BH_4_ alone”, open bars), 6RBH_4_ plus probenecid, 200 mg/kg, i.p. (“BH_4_ + Pbc”, closed bars), or 6RBH_4_ plus cyclosporin A, 10 mg/kg, i.p. (“BH_4_ + CsA”, grey bars). The open bars on the left represent the results of “BH_4_ alone” in experiments performed at the same time with “BH_4_ + Pbc”, and the open bars on the right represent “BH_4_ + CsA” at the respective time points. In all panels, the rat group “before” received no treatment in all respective drug-administered groups. “BP” denotes BH_2_ + BH_4_. The calculation of the amount of BP per volume of original blood (mL) was based on the hematocrit value. Data are means ± S.E. (n = 4 in “BH_4_ + Pbc” and n = 5 in “BH_4_ + CsA”). Student's *t*-test, “BH_4_ alone” *vs*. “BH_4_ + Pbc”, or “BH_4_ alone” *vs*. “BH_4_ + CsA”, *P<0.05, **P<0.01.

The initial BP level in the plasma was 0.13 ± 0.02 nmol/mL. After rats were administered 6RBH_4_, the plasma BP of all groups, “BH_4_ alone”, “BH_4_ + Pbc” and “BH_4_ + CsA”, was 30- to 50-fold over the initial value (Fig 3A). The elevated BP level in “BH_4_ + Pbc” rats at 30 min was 1.8-fold higher than that in “BH_4_ alone” (*P*<0.05). The difference between BP levels in “BH_4_ + CsA” and “BH_4_ alone” was not clear. Plasma BP levels in both groups of rats returned to around 1 nmol/mL at 120 min after 6RBH_4_ administration but they were about 10-fold greater than the initial value. The initial BP level in RBCs was higher than in plasma (0.43 ± 0.06 nmol/mL) and roughly 77% of the blood BP was localized in these cells. The amount of BP in RBCs changed more slowly than that in plasma (Fig 3A and 3B).

### Increase in BP contents in the liver, kidney and brain

**Fig 4:**
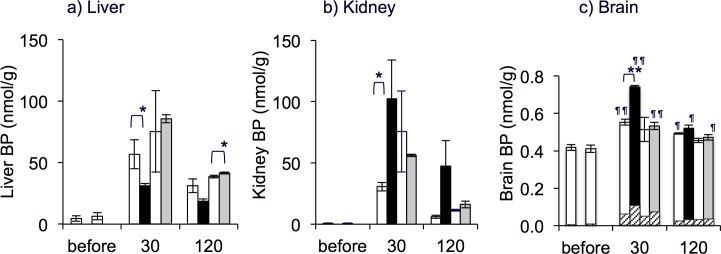
Organ distribution of biopterin after 6RBH_4_ administration and the effect of probenecid and cyclosporin A. BP levels in the liver (a), kidney (b), and brain (c) were determined. Rats were given 6RBH_4_ alone (5 mg/kg, i.v., “BH_4_ alone”, open bars) or 6RBH_4_ with prior administration 30 min earlier of probenecid (“BH_4_ + Pbc”, 200 mg/kg, i.p., closed bars) or cyclosporin A (“BH_4_ + CsA”, 10 mg/kg, i.p., grey bars). The open bars on the left represent the results of “BH_4_ alone” in experiments performed at the same time as with “BH_4_ + Pbc”, and the open bars on the right were those with “BH_4_ + CsA” at respective time points. The hatched bars in (c) indicate blood carryover of biopterin based on estimated blood inclusion (1.2%) in the brain. After the indicated periods, rats were sacrificed, and the organs were dissected as described in experimental procedure (B) of Materials and Methods. Samples were taken from the same rats as those in Fig 3. Student’s *t*-test (“BH_4_ alone” *vs*. BH_4_ + drug at respective time points), *P<0.05; Tukey’s test (“before” *vs*. 30 or 120 min), ^¶^*P*<0.05, ^¶¶^*P*<0.01; data are means ± S.E., (n = 4 for Pbc series, n = 5 for CsA series).

The BP contents increased in the liver, kidney and brain after 6RBH_4_ administration as shown in Fig 4. The initial BP levels in the organs were as follows: liver, 5.86 ± 0.47; kidney, 0.68 ± 0.04; and brain, 0.41 ± 0.01 nmol/g. The protocol used was experimental procedure (B) in the Materials and Methods section.

#### Liver

The liver took up a large amount of BP within the short term; the BP content increased to 53.3 nmol/g (9.1-fold over the initial value) in the “BH_4_ alone” rats within the first 30 min (Fig 4A). With Pbc and CsA, the effect was different; the increase in the “BH_4_ + Pbc” group (5.3-fold) was considerably less than in the “BH_4_ alone” group (*P*<0.05 at 30 min), while that of “BH_4_ + CsA” was rather increased (*P*<0.05 at 120 min). Two interesting findings emerged: 1) the net uptake by the liver was suppressed considerably, 9.1-fold down to 5.3-fold, even though the elevation of the plasma BP doubled in the presence of Pbc (Fig 3A), suggesting that the rapid uptake was strongly inhibited to nearly one quarter by the drug. This was in sharp contrast to “BH_4_ + CsA” rats in which the BP accumulation was elevated to a level corresponding to that in the blood, suggesting that CsA did not inhibit BP uptake in this organ. 2) the release process was not greatly inhibited by the drugs either Pbc or CsA; the decrease in liver BP levels in the three groups seemed proportional over the 30–120 min period. If the BP uptake was mediated by a transporter, the fact that Pbc inhibited BP uptake by the liver suggested that the putative transporter was Pbc-sensitive and was somehow inward-directional for BP transport from the blood. BP uptake by the liver in comparison to uptake by the other organs is summarized in Table 1.

**Table 1:** Biopterin distribution in organs after 6RBH_4_ administration.

			0-time		30 min		120 min	
	organ weight (g)	relative^1)^to body	BP (nmol)	relative^2)^to liver	BP (nmol)	relative^3)^to dose	BP (nmol)	relative^3)^to dose
Liver	**6.9**	2.8%	**40.4**	(100%)	**367**	7.1%	**268**	5.2%
Blood	**17**	6.8%	**8.32**	21%	**76.8**	1.5%	**39.5**	0.76%
Kidneys	**1.8**	0.72%	**1.22**	3.0%	**70.6**	1.4%	**17.3**	0.3%
Brain	**1.5**	0.60%	**0.62**	1.5%	**0.8**	0.15%	**0.71**	0.14%
Sub-total	**27**	11%	**50.6**	n.a.	**515**	9.9%	**325**	6.3%

Biopterin distribution in liver compared with the other organs. Data apply to Figs 3 and 4. BP contents (nmol/organ) are expressed as the mean values and are multiplied by the respective organ weights (g).

1) Relative to a 250 g body weight, adapted from “SD-Rat Control Data 2009” of Charles River Laboratories Japan, Inc.

2) Relative to the amount of BP in whole liver (nmol)

3) Relative to dose: (increase in organ BP over 0-time) / (5180 nmol: 6RBH_4_ dose per 250 g).

#### Kidney

Although the endogenous BP in the kidney was only 3% of that in the liver, this organ had a relatively high capacity to uptake exogenous BP. The amount of tissue BP was greatly increased (58-fold over the initial value) in the “BH_4_ alone” rats at 30 min (Fig 4B). The BP rise in the “BH_4_ + Pbc” rats was even greater (150-fold), as was the rise in “BH_4_ + CsA” rats (about 80-fold), both corresponding to the respective rises in the plasma BP (compare Fig 4B with Fig 3A). The decrease in BP in all three groups during the period between 30 and 120 min was quite rapid, similar to the change noted in plasma. Taking into account that at 30 min, the peak amount of BP in the kidney was only 4–5% of the urinary BP outflow, in which renal tubular secretion was dominant as mentioned earlier (cf. Fig 1C), it is reasonable to assume that the BP in kidney tissue transited from the plasma to the urine across the tubular epithelium. This is in remarkable contrast to the liver BP which was stored in a large quantity then released gradually into the plasma.

#### Brain

The brain BP was significantly increased in all three groups, although much less than that of the other organs (Fig 4C). The BP contents of “BH_4_ alone” rats were about 43% higher after 30 min than those of untreated rats at (*P*<0.01). At this time point, the increase in “BH_4_ + Pbc” rats was significantly larger, as much as 65% (*P*<0.01), compared to that of “BH_4_ alone” rats (*P*<0.05). Since the blood content included in the brain samples was about 1.2% (v/w), it was uncertain whether the increase was caused by a blood carryover which contained increased the amount of exogenous BP (cf. Fig 3C). The BP carryover was calculated and compared with the respective BP contents of each group using mean values of BP in time-matched blood (Fig 4C). When the observed increases in BP were adjusted by subtracting the carryover, the net increase in the “BH_4_ alone” rats for the first 30 min was roughly 15%. Similarly, in “BH_4_ + Pbc” and in “BH_4_ + CsA” rats, BP levels were elevated by 41% and 13%, respectively. Therefore, a greater increase in BP content was verified in “BH_4_ + Pbc” rats than in “BH_4_ alone” rats (*P*<0.05).

The increase in the brain BP observed in this study might not be sufficient for stimulation of monoamine biosynthesis as reported [20, 21]. In fact, Miwa et al [22] reported that a 50- to 200-fold increase in BH_4_ was needed, when the preparation was injected directly into the brain ventricle for significant stimulation of monoamine biosynthesis. Although the elevation observed in this study seems to be insufficient for stimulating the biosynthesis of monoamines, it could be beneficial for neural stimulation of their release [23].

### The BH_4_ fraction (BH_4_%) in organ biopterin and the effect of probenecid

**Fig 5:**
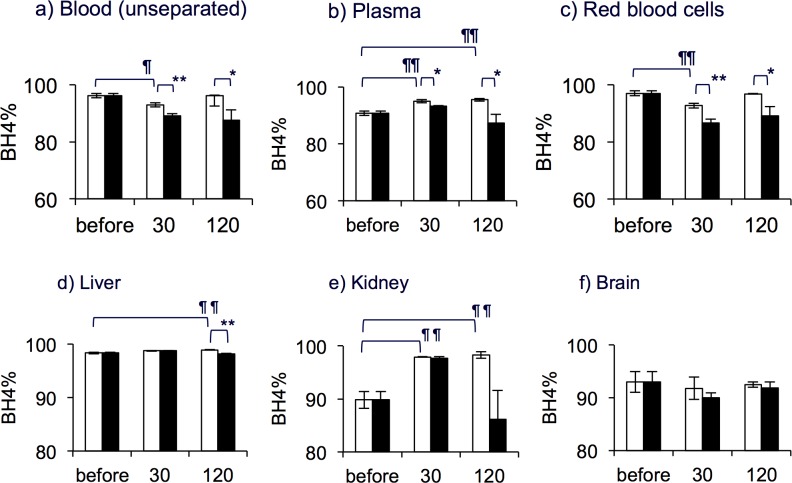
Effect of probenecid on the BH_4_ fraction (BH_4_%) in blood biopterin. Panels show levels in whole blood in which the plasma and RBCs were not separated (a), plasma (b) and RBCs (c), liver (d), kidney (e) and brain (f). Rats were given 6RBH_4_ alone (5 mg/kg, i.v., “BH_4_ alone”, open bars) or 6RBH_4_ with prior administration 30 min earlier of probenecid (“BH_4_ + Pbc”, 200 mg/kg, i.p., closed bars). In all panels, the “before” rat group did not receive either 6RBH_4_ or Pbc. The experimental procedure was the same as described in Figs 3 and 4. The BH_4_% was determined as described in Materials and Methods. Data are means ± S.E. (n = 4). Tukey’s test for multiple comparison, “before” *vs*. 30 min and 120 min, ^¶^*P*<0.05, ^¶¶^*P*<0.01. Student’s *t*-test for two groups, “BH_4_ alone” *vs*. “BH_4_ + Pbc”, *P<0.05, **P<0.01.

Alterations in the BH_4_% along with the BP distribution profiles in respective organs (Figs. 3 and 4) are depicted in Fig 5.

The BH_4_% in untreated whole blood, on which the plasma and RBCs were not separated, was 96.2 ±0.2%, and it decreased significantly along with time (Fig 5A, *P*<0.05). Although the BH_4_% in the plasma showed a significant but slight increase at 30 and 120 min, the lowering of the BH_4_% in RBCs was relatively large (Fig 5C, *P*<0.01) and pulled the blood BH_4_% down. All samples, plasma, RBCs as well as the unseparated blood, taken from “BH_4_ + Pbc” rats showed a significantly lower BH_4_% than those of “BH_4_ alone” rats.

The BH_4_% in the liver, kidney and brain are shown in Fig 5D–5F. The initial BH_4_% in these organs were: liver, 98.3 ±0.1%; kidney, 89.8 ±1.6%; and brain, 93.0±2.0%. In the liver, the BH_4_% was quite stable at levels higher than 98% in all groups. In the presence of Pbc, however, namely in “BH_4_ + Pbc” rats, the BH_4_% was lowered slightly but significantly at 120 min (Fig 5D, “BH_4_ alone” *vs*. “BH_4_ + Pbc”, *P*<0.01). The kidney BP showed a considerable increase in its BH_4_% after the 6RBH_4_ administration (Fig 5E, “before” as the reference, *P*<0.01). It was noted that profiles of the BP content in the blood and kidney looked quite similar (compare Fig 3A and Fig 4B) in the period after the 6RBH_4_ administration. The brain took up a small amount of blood BP as seen in Fig 4C, and no significant change in the BH_4_% was observed in this study (Fig 5F). The significance of correlation between the BH_4_% of blood, liver and kidney is listed in Table 2. In this Table, the BH_4_% of all organs after 6RBH_4_ administration with or without Pbc or CsA are listed and compared with respect to the statistical significance of differences between the organs. The BH_4_% in the liver and kidney was persistently higher than that of blood BP, while the data showed no statistically significant difference between those of liver and kidney.

**Table 2:** The BH_4_% in the blood, liver and kidney after 6RBH_4_ administration.

Time	Rat group	Blood	Liver ^1)^		Kidney ^1)^^,^ ^2)^	
		%	%		%	
30 min	BH_4_ alone	93.9 ±0.6	98.7 ±0.1	**	98.0 ±0.2	**, n.s.
	BH_4_ + Pbc	89.2 ±0.7	98.7±0.1	**	97.7 ±0.3	**, n.s.
	BH_4_ + CsA	94.4 ±0.1	98.6 ±0.1	**	98.1 ±0.4	**, n.s.
120 min	BH_4_ alone	96.2 ±0.1	98.8 ±0.1	**	98.6 ±0.3	**, n.s.
	BH_4_ + Pbc	87.6 ±3.7	98.1 ±0.1	n.s.	89.2 ±5.5	n.s., n.s.
	BH_4_ + CsA	95.3 ±0.1	98.5 ±0.1	**	98.4 ±0.1	**, n.s.

The BH_4_% in organs 30 min and 120 min after administration of 6RBH_4_ with or without prior treatment with Pbc or CsA. For each rat group, differences in the organ BH_4_% between blood, liver and kidney were analyzed by Tukey’s multiple testing method. BH_4_% values are means ±S.E. and apply to Fig 5.

^1)^ Difference between the BH_4_% of time-matched blood *vs*. liver or kidney. **P<0.01 significance, suggesting the BH_4_% is higher than that of blood; “n.s.”, not significant.

^2)^ Difference between the BH_4_% of time-matched liver *vs*. kidney.

## Discussion

### Urinary excretion was the major route of BP loss while bile was a minor route

Renal exclusion of BH_4_ immediately after 6RBH_4_ administration was remarkably rapid. The major portion of the exclusion, up to ca. 87% of the apparent elimination observed within 270 min, took place in the first 120 min in healthy rats. Kidney exclusion of xenobiotics and metabolic waste generally takes two paths from plasma to urine; glomerular filtration and tubular secretion [24]. The tubular secretion of exogenously administered BH_4_ was effectively suppressed by prior administration of Pbc, as shown in this report, providing confirmative evidence supporting a previous observation using CsA [12, 13]. The previous report demonstrated that the urinary BP flow involved at least two processes, glomerular filtration with a rate constant of k = 0.013 min^-1^, and tubular secretion with a 2.3-fold more rapid rate constant of k = 0.030 min^-1^, which represented the difference between the observed gross rate constant of k = 0.043 min^-1^ minus the glomerular rate constant. The present data on “BH_4_ + Pbc” rats after 90 min (Fig 1B, lower panel) also fitted the equation *BP(t)* = 9.76 • e^−0.012*t*^ (|R| = 0.92), suggesting that the urinary flow was essentially from glomerular filtration at 90 min or later after 6RBH_4_ administration. The treatment with Pbc greatly increased the blood BP by inhibiting BP uptake by various organs including the kidney. Despite the substantial suppression, BP exclusion within 270 min after 6RBH_4_ administration was almost the same in “BH_4_ alone” and “BH_4_ + Pbc” rats. This was explained by the fact that the concomitantly high concentration of plasma BP caused by the Pbc treatment speeded up the glomerular filtration. It should be noted that the tubular secretion was enhanced when mediated by a drug-sensitive transporter(s) presumably with large capacity but low affinity. This infers that the putative transporter only functions when the plasma BP concentration is high. Actually, it was demonstrated that the dominancy of epithelial secretion over glomerular filtration occurred only when the plasma BP concentration was 10 times higher than ordinary levels [12, 13]. In this context, adjusting the formula or recipe for 6RBH_4_ administration so as to avoid the tubular secretion by maintaining the plasma BP level around the critical concentration (~ 10-fold) may result in a much higher efficiency in bodily replacement of BH_4_.

We previously described excretion of BP in the bile as a route for its entero-hepatic circulation [14]. In the present experiment following experimental procedure (A) in the Materials and Methods section, we removed all the bile and did not let it return to the liver. After 6RBH_4_ administration, the bile BP increased along with the liver BP levels in the presence or absence of Pbc. Further, the BH_4_% of the bile BP was persistently high, similar to the high BH_4_% in the liver, either in the presence or absence of Pbc. Pbc might have inhibited BP uptake by the liver cells but might not have affected its release into the bile. We also noted that the cumulative excretion of BP in the bile was only about 3% that in the urine. Although it was reported that biliary excretion was a major route for the elimination of various drugs or their metabolites [24, 25], our findings revealed that it was rather a minor route with regard to the bodily loss of administered BH_4_. However, the fact that the liver excels in secreting BP with a high BH_4_% suggests that it has some role in BH_4_ secretion into the gut, however, the physiological function of the bile BH_4_ remains elusive.

### Distribution and retention of administered 6RBH_4_ in the organs

Hoshiga *et al*. [26] described the body-wide distribution of exogenous BH_4_ which they detected by means of whole body autoradiography after i.v. injection of a tracer amount of (6R)-[U-^14^C]BH_4_. They found the densest deposition of radioactivity to be in the kidney at 2 hours. Among large organs, the liver had the next densest deposition, while deposition in the brain and striated muscles was unremarkable. Hayashi *et al*. (in a Japanese journal [27]) described BP distribution after administration of 6RBH_4_. According to their report, the BP contents of various organs including the liver were all highest at 30 min (the earliest sampling) and the BP amount in each organ decreased gradually over the course of the 24-hour experiment, suggesting that the uptake phase of BP, in terms of the net balance of uptake minus release, had been virtually completed in organs within the first 30 min. In our present research, we treated rats with Pbc or CsA prior to administration of 6RBH_4_ with the purpose of examining the effect of these inhibitors on BH_4_ uptake by various organs and the respective differences in the BH_4_%. We started sampling at 30 min, assuming that the net uptake had already been completed by the time that BP was circulating throughout the body based on the data reported by Hayashi *et al*. above. The retention of the BP accumulated in the liver was longer than that of the kidney and brain, and that of RBCs in the blood. Taking its large mass into account, the liver seemed to function as a large reservoir of the administered BH_4_ for gradual supply to peripheral tissues including the vasculature. This meant that, over the long period, the liver released BP gradually to the bile and presumably to the blood circulation.

### Homeostasis of BH_2_/BH_4_ after administration of 6RBH_4_

We observed that the BH_4_% in the blood and urine was considerably decreased while the amount of tissue BP was prominently increased in the liver and kidney after 6RBH_4_ administration. Further, Pbc, an uptake inhibitor, enhanced the BH_4_% decrease in the blood and urine. These results raised two questions: first, why did the BH_4_% decrease after 6RBH_4_ administration and secondly, why did the uptake inhibitor promote a further decrease.

As to the first question, we consider that the answer lies in the mechanisms by which cells take up extracellular BH_4_. BH_4_ is barely able to cross the cell membrane in its tetrahydro-form because as an active coenzyme, it must remain inside the cell. Generally, the cell membrane works as a strong barrier against the in and out movement of such a hydrophilically active coenzyme as BH_4_. For the uptake of extracellular BH_4_, a complex series of events must occur [28]. First, BH_4_ is oxidized, the resultant BH_2_ is taken up by the cell, an action presumably mediated by transporters, and then BH_2_ is reduced back to BH_4_ through the salvage pathway. Although the location and manner of the rapid oxidation of the administered 6RBH_4_ was ambiguous, the systemic oxidation of BH_4_ to BH_2_ *in vivo* was clearly demonstrated by the appearance of a BH_2_ serge in the blood immediately after 6RBH_4_ administration in the mouse [19]. In the present study using rats under anesthesia, we did not observe such a prominent BH_2_ surge after 6RBH_4_ administration, but noted instead a significant decrease in the BH_4_%, in other words, an increase in BH_2_ relative to BH_4_, in the blood and urine. The moderate but long-lasting decrease in the BH_4_% in rats seemed to result from essentially the same process responsible for the rapid appearance of the BH_2_ serge in mice. We consider that in both rats and mice, these outcomes reflect an inevitable compensatory response of the animal body toward exogenous BH_4_.

As for the second question, the answer is contained in the response to the first. The reducing action of BH_2_ is an intracellular event but the BH_2_ has to be brought into the cell from the plasma. Therefore, BH_2_ uptake by the tissues is a prerequisite for scavenging blood BH_2_. Although the uptake transporters have not all been identified, at least we know that two equilibrative nucleoside transporters called ENT1 (SLC29A1) and ENT2 (SLC29A2) are able to mediate BH_2_ relocation favoring the other dihydropterin-compound, sepiapterin, but not BH_4_ [29]. Some portion of the BP uptake observed in this study was obviously mediated by another transporter(s) because ENT1 and ENT2 are sensitive to nitrobenzylthioinosine (NBMPR) but not to Pbc to our knowledge. The fact that the blockade of BP uptake by Pbc enhanced a further decrease in the blood BH_4_% suggests that BH_2_ was effectively or even preferentially taken up by the putative transporter(s) which was sensitive to Pbc. Since the salvage pathway is thermodynamically favored to proceed, the internalized BH_2_ is readily scavenged as in the push-pull accumulation of BH_4_ [18, 30]. As for extracellular BH_4_, however, we have not uncovered any form of active transport that would allow it to directly accumulate in the cell against a concentration gradient in a tetrahydro-form. A passive transport could not contribute to BH_4_ accumulation against the concentration gradient even if a transporter(s) could potentially move it across the plasma membrane.

The endogenous content and the capacity to uptake BH_4_ was prominent in the liver and kidney (Table 1). This suggested to us that most BH_2_ scavenging might have occurred in these organs. It was noted that the BH_4_% in these organs did not decrease when the BH_4_% was lowered in the blood and urine after 6RBH_4_ administration. BP uptake by the liver was considerably inhibited by prior treatment with Pbc and it was accompanied by an even greater decline in the BH_4_% of the blood and urine. This observation was explained by inhibition of the uptake process by the drug and the consequent inability of the liver to convert BH_2_ to BH_4_. Taking the liver’s large capacity for BP uptake into account, this organ might have played the greatest role in removing the scavenged BH_2_.

Considering that the kidney excreted urine with a consistently higher BH_4_% than that of the plasma, the strong ability of the kidney to scavenge BH_2_ was also noted. The pronounced decrease in the BH_4_% in the urine in the presence of Pbc suggested reduced BH_2_ scavenging in the kidney. This Pbc sensitivity indicates that the BH_2_ scavenging occurred during the transit across the tubular epithelium for secretion. In this manner, the kidney was able to scavenge BH_2_ from the plasma. Additionally, the kidney might have released a large amount of BH_2_-scavenged “clean” BP back to the plasma resulting in a rise in the plasma BH_4_%, but our experiment was not designed to show this explicitly. The details of tubular secretion with regard to the relevant transporter for BH_2_ scavenging remain elusive.

When our focus was limited to classic enzyme pathways, the salvage pathway of BH_4_ was seen as a mechanism for maintaining BH_4_ levels against a loss of BH_4_ cofactor activity. The salvage pathway, coupled with uptake of the extracellular precursor, should now be considered as an innate mechanism for scavenging BH_2,_ a mechanism which covers a wide range of interactions between cells and their environment throughout the body. The liver and kidney are the major sites of BH_2_ scavenging under conditions of 6RBH_4_ administration owing to their large-scale uptake of blood BP.

### Remarks concerning NOS dysfunction

In clinical practice, 6RBH_4_ has been prescribed to patients with an inherited BH_4_ deficiency as well as to many with BH_4_-responsive phenylketonuria. On a search of the literature, we did not encounter any mention of a serious adverse effect of long-term administration in these patients [31]. In another area of medicine, BH_4_ supplementation has also drawn increased attention with respect to whether it ameliorates NOS dysfunction in the cardiovascular system as a high BH_2_/BH_4_ ratio, representing a type of oxidative stress, is a great risk factor. Studies in this area are concerned with the homeostasis of the BH_2_/BH_4_ ratio, an inverse expression of the BH_4_%.

The administration of 6RBH_4_ is followed by a lowering of the blood BH_4_% as an inevitable compensatory reaction, as discussed above. In the case of the lowering of the BH_4_% in the blood, endothelial cells are the ultimate interface in all organs and are active sites of nitric oxide (NO) production regulating vascular contraction. In fact, we previously demonstrated that ENT1 and ENT2 were able to mediate cell membrane permeation of sepiapterin preferentially, permeation of BH_2_ moderately and that of BH_4_ only slightly [29]. Furthermore, we localized ENT2 on the vascular lumen side of endothelial cells [32], suggesting that these cells are ready to uptake increased amounts of BH_2_. Owing to two characteristic properties of the enzyme NOS, once in the cell, BH_2_ must be converted to BH_4_ promptly. 1) The affinity of NOS for BH_2_ is not particularly low compared to its affinity for BH_4_; in the case of an nNOS, an isoform of eNOS, the affinity to BH_2_ and to 6RBH_4_ was as close as 1: 10 [33]. 2) BH_2_-bound NOS is unable to maintain an active dimer and uncouples O_2_ reduction to produce superoxide [34–36]. In this context, dihydrofolate reductase is the key enzyme for maintaining a BH_2_/BH_4_ ratio sufficiently low for sound NO production. However, endothelial cell-derived culture cells, especially of human origin, are poorly furnished with dihydrofolate reductase [37–39], suggesting that the original endothelial cells in the human vasculature have the same vulnerability. Consequently, a high BH_2_/BH_4_ ratio in the plasma causes eNOS to uncouple locally in endothelial cells and leads to a negative spiral favoring BH_4_ oxidation [40]. In this study, we noticed that after 6RBH_4_ administration, the blood BH_4_% persistently dropped below 90%, that is, the BH_2_ to BH_4_ ratio became less than 1:10 even in the “BH_4_ alone” group, while it was maintained at ca. 95% in untreated rats. We also noted that Pbc, a widely prescribed anti-gout drug, further increased the blood BH_2_ when used with 6RBH_4_. It is interesting that many experimental studies support the use of 6RBH_4_ administration as a means of ameliorating cardiovascular symptoms, however, translation of these studies to human patients remains limited [41]. The stated vulnerability of endothelial cells suffering from a lowering of the blood BH_4_% might represent a great risk factor for possible exacerbation of cardiovascular dysfunction in cases in which 6RBH_4_ is regularly administered repeatedly at a dose of 5–20 mg/kg/day.

We conclude that supplementation with BH_4_ by means of administration of 6RBH_4_ has remarkably low efficacy due to its rapid loss through the urine, nonetheless it is still effective in raising BH_4_ levels in peripheral organs. However, considering the general vulnerability of the cardiovascular system to an increasing BH_2_/BH_4_ ratio, regular administration of 6RBH_4_ may spoil the desired effect of countering NOS dysfunction by lowering the BH_4_%. The compensatory lowering of the BH_4_% must be avoided in future improvements to the therapeutic composition. Take together improving 6RBH_4_ preparations to allow for sustained distribution while avoiding a steep elevation in plasma might help to realize the potential benefit of BH_4_ supplementation.
